# Polymer link breakage of polyimide-film-surface using hydrolysis reaction accelerator for enhancing chemical–mechanical-planarization polishing-rate

**DOI:** 10.1038/s41598-022-07340-y

**Published:** 2022-03-01

**Authors:** Gi-Ppeum Jeong, Jun-Seong Park, Seung-Jae Lee, Pil-su Kim, Man-Hyup Han, Seong-Wan Hong, Eun-Seong Kim, Jin-Hyung Park, Byoung-Kwon Choo, Seung-Bae Kang, Jea-Gun Park

**Affiliations:** 1 Department of Nanoscale Semiconductor Engineering, Hanyang University, Seoul, 04763 Republic of Korea; 2 Department of Electronics and Communications Engineering, Hanyang University, Seoul, 04763 Republic of Korea; 3 UB Materials Inc., Gyeonggi-do, 17162 Republic of Korea; 4 Department of Process Research Team, Display Research Center, Samsung Display Co., Ltd., Gyeonggi-do, 17113 Republic of Korea

**Keywords:** Chemical engineering, Electrical and electronic engineering, Mechanical engineering

## Abstract

In this study, the chemical decomposition of a polyimide-film (i.e., a PI-film)-surface into a soft-film-surface containing negatively charged pyromellitic dianhydride (PMDA) and neutral 4,4′-oxydianiline (ODA) was successfully performed. The chemical decomposition was conducted by designing the slurry containing 350 nm colloidal silica abrasive and small molecules with amine functional groups (i.e., ethylenediamine: EDA) for chemical–mechanical planarization (CMP). This chemical decomposition was performed through two types of hydrolysis reactions, that is, a hydrolysis reaction between OH^−^ ions or R-NH_3_^+^ (i.e., EDA with a positively charged amine groups) and oxygen atoms covalently bonded with pyromellitimide on the PI-film-surface. In particular, the degree of slurry adsorption of the PI-film-surface was determined by the EDA concentration in the slurry because of the presence of R-NH_3_^+^, that is, a higher EDA concentration resulted in a higher degree of slurry adsorption. In addition, during CMP, the chemical decomposition degree of the PI-film-surface was principally determined by the EDA concentration; that is, the degree of chemical composition was increased noticeably and linearly with the EDA concentration. Thus, the polishing-rate of the PI-film-surface increased notably with the EDA concentration in the CMP slurry.

## Introduction

In general, the material properties of a polyimide include low stress, low thermal coefficient of expansion, low moisture, high modulus, good ductility, thermal stability, high glass transition temperature, and low process cost^1–20^. Thus, recently, polyimide has been intensively applied in semiconductors for an interlayer dielectric polymer for thin-film metallization and a flexible and low-stress polymer for wafer-level packaging^6–18^. In addition, the use of polyimide films as interlayer dielectrics and flexible substrates in flexible display have significantly increased in recent years^19–30^. Moreover, polyimide is also used as a gap-filling polymer in microelectromechanical systems (MEMS)^5^. These applications of polyimide essentially require chemical–mechanical planarization (CMP) of the polyimide film surface^1–4^. In the last decade, only mechanically dominant CMP of the polyimide film has been conducted because a polyimide film surface is a hard covalently bonded material, performing a low polishing-rate^1–4^. Generally, a polyimide film has been composed of a polymer of pyromellitic dianhydride (PMDA) linked with 4,4′-oxydianiline (ODA), including strong covalent C–C (i.e., 285.0 eV), C=C (i.e., 284.5 eV), C=O (i.e., 532 eV), and C–N (i.e., 286.2 eV in binding energy) bonds. Thus, only mechanical polishing of the polyimide film surface could perform a relatively low polishing rate, since any organic chemical added in the PI-film CMP slurry is extremely difficult to be chemically reacted with the PI-film CMP slurry^1–4^. As a solution, a drastic surface deformation of the polyimide film surface from a hard to a soft film surface is essentially required for enhancing chemically dominant CMP rather than mechanically dominant CMP. In our study, we designed a novel hydrolysis reaction accelerator in the polyimide-film surface CMP slurry, breaking a link between PMDA and ODA in the polyimide film rather than breaking C–C, C=C, C–O, and C–N bonds in the polyimide film surface. In particular, the design of a hydrolysis reaction accelerator and a polyimide-film-surface CMP slurry should be considered for enhancing the chemically dominant CMP. Therefore, in this study, an accelerator for a novel hydrolysis reaction was designed with small molecules containing amine functional groups (i.e., ethylenediamine: EDA) that could be converted to positively charged amine functional groups (i.e., R-NH_3_^+^) and generated a high concentration of a negatively-charged hydroxide (i.e., OH^-^) in the polyimide film surface CMP slurry. As a result, both R-NH_3_ and OH^-^ can participate significantly the breakage of a link between PMDA and ODA, transforming a hard to a soft polyimide film surface, which will be proven evidently. First, the effect of the polyimide film surface polishing-rate on the EDA concentration in the slurry was estimated as a function of the slurry properties. In addition, the slurry adsorption degree on the polyimide film surface was evaluated by measuring the slurry contact angle on the polyimide film surface as a function of the EDA concentration in the CMP slurry. The presence of carboxyl functional groups on the polyimide film surface after CMP was investigated by X-ray photoelectron spectroscopy (XPS). This investigation revealed the chemical decomposition of a hard polyimide film surface into a soft film surface containing pyromellitic dianhydride (PMDA) and neutral 4,4′-oxydianiline (ODA). In particular, the CMP-induced decomposition mechanism of the polyimide film surface was clearly characterized by two types of hydrolysis reactions between OH^–^ or R-NH_3_^+^ and the polyimide film surface. Finally, the decomposition degree of the polyimide film surface with respect to the EDA concentration was evaluated during the CMP.

## Results

### Dependency of PI-film polishing-rate on the concentration of hydrolysis reaction accelerator with amine functional groups

**Figure 1:**
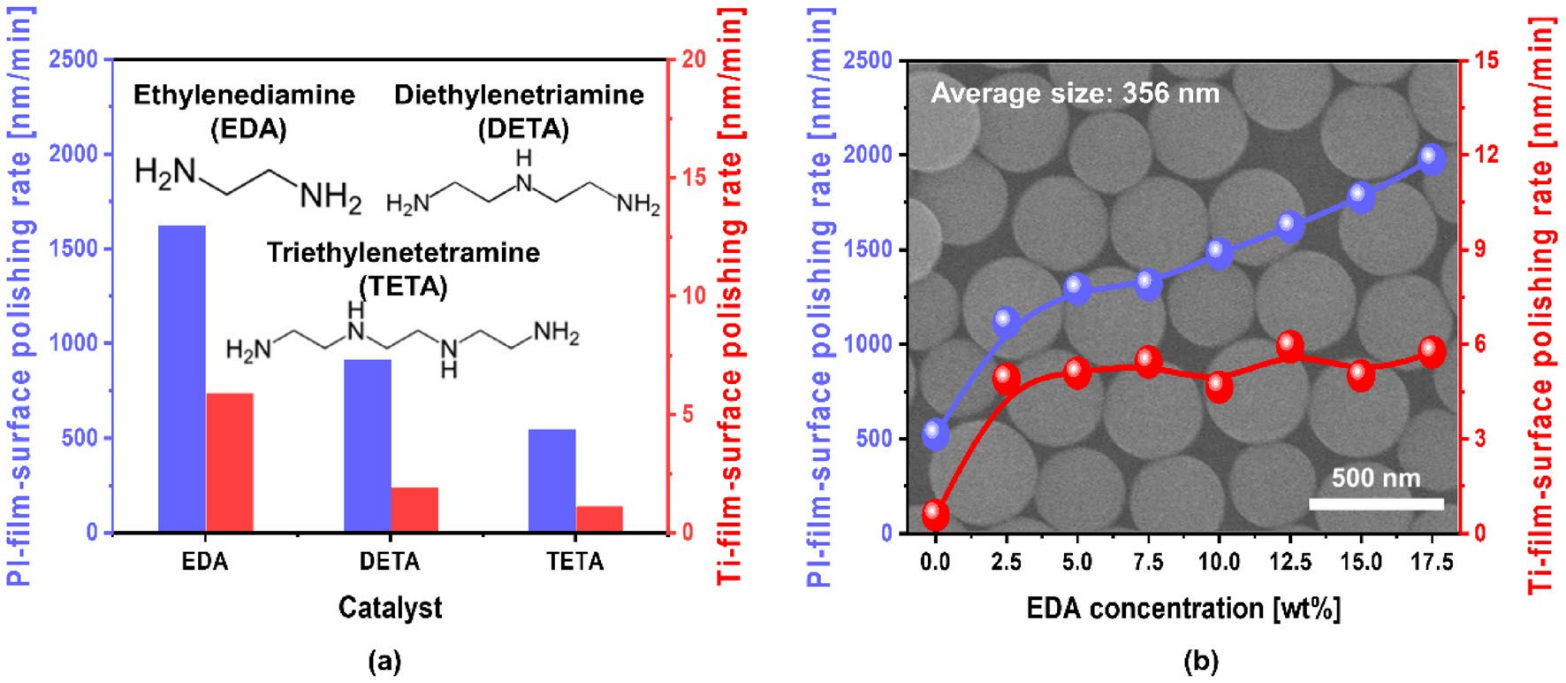
Effect of a hydrolysis reaction accelerator on the PI-film polishing-rate. (**a**) Polishing-rates of PI- and Ti-films in the presence of different hydrolysis reaction accelerators such as EDA, DETA, and TETA. The number of amine functional groups on EDA, DETA, and TETA are 2, 3, and 4, respectively. (**b**) Dependency of the PI- and Ti-film polishing-rates on EDA concentration in the PI-film CMP slurry. The SEM image (i.e., background image of (**b**) of the slurry indicated that the average size of the colloidal silica abrasives in the slurry was 350 nm.

To enhance the polishing-rate of a PI-film, a hydrolysis reaction on the film surface to chemically decompose the harder PI-film-surface into a soft PI-film-surface is essential during the CMP process. Small molecules with amine functional groups could enhance the polishing-rate of the PI-film-surface. Therefore, three types of small molecules with amine functional groups were tested: EDA, diethylenetriamine (DETA), and triethylenetetramine (TETA), as shown in Fig. 1a. As a reference, a slurry containing 3 wt% colloidal silica abrasives, with particle diameter of 350 nm, was prepared at pH 9.2, and the PI-film polishing-rate of the slurry was ~ 500 nm/min. In contrast, the CMP slurries containing 3 wt% colloidal silica abrasives (particle diameter = 350 nm) and the hydrolysis reaction accelerators such as EDA, DETA, and TETA exhibited PI-film polishing-rates of ~ 1600, 900, and 540 nm/min and Ti-film polishing-rates of ~ 6, 2, and 1 nm/min, respectively. Thus, EDA was selected as a hydrolysis reaction accelerator for a PI-film CMP slurry to enhance the hydrolysis reaction in the PI-film-surface. In addition, the dependency of the PI-film polishing-rate on the EDA concentration in the CMP slurry was investigated, as shown in Fig. 1b. When the EDA concentration increased from 0 to 17.5 wt%, the PI-film polishing-rate linearly and significantly increased from 500 to 2000 nm/min, while the Ti film polishing-rate slightly increased from 0.5 to 6 nm/min and saturated thereafter. This result indicates that a hydrolysis reaction accelerator, such as EDA, enhances the PI-film polishing-rate by introducing a hydrolysis reaction on the PI-film-surface during the CMP, which will be proven later in detail.

### Effect of CMP slurry properties on the PI-film polishing-rate

**Figure 2:**
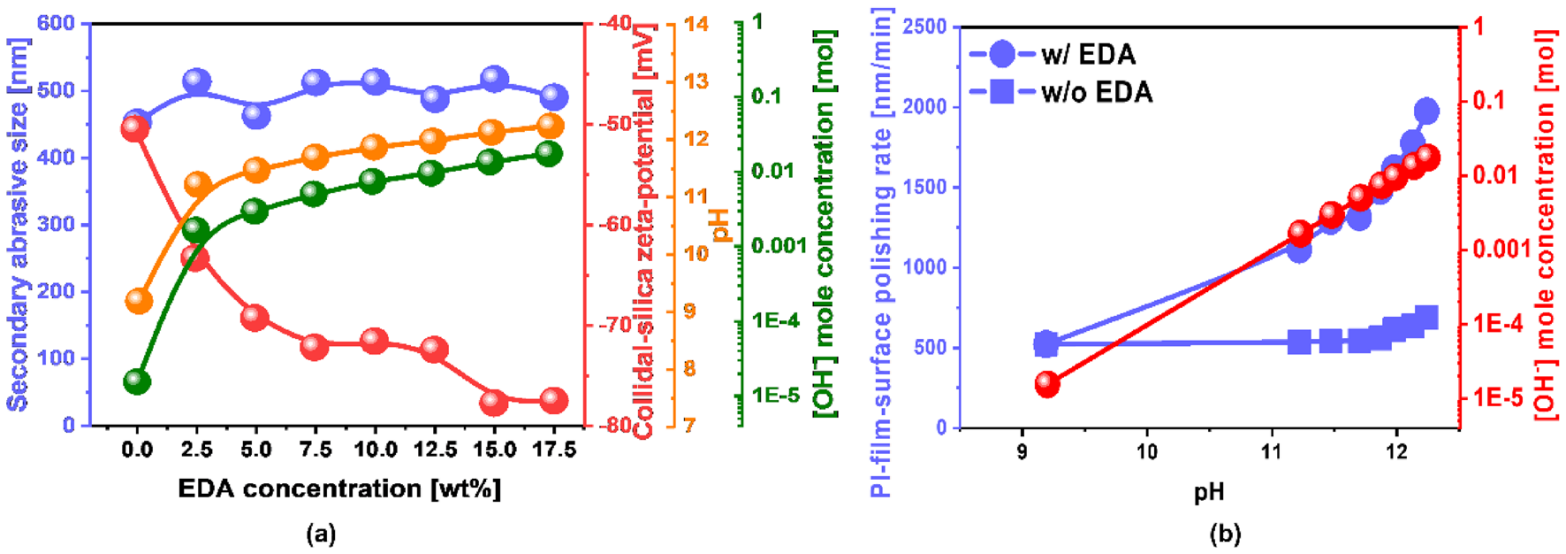
Correlation between CMP slurry properties and the PI-film polishing-rate. (**a**) Dependency of the secondary abrasive size, colloidal silica abrasive zeta potential, slurry pH, and OH^−^ mole concentration on EDA concentration in the slurry. (**b**) Comparison between the OH^−^ mole concentration effect and the EDA concentration effect on enhancing the PI-film polishing-rate. The slurry pH in the absence of EDA was adjusted by using a titrant (i.e., KOH), while the slurry pH in the presence of EDA was mediated by using a hydrolysis reaction accelerator (i.e., EDA).

To understand the dependency of the PI-film polishing-rate on the EDA concentration, the slurry properties such as the secondary size and zeta potential of the colloidal silica abrasives, pH, and OH^−^ mole concentration were observed as a function of the EDA concentration, as shown in Fig. 2a. The colloidal silica abrasive secondary size increased slightly from 452 to 512 nm immediately upon the addition of EDA. It slightly increased with the EDA concentration since the chemical double layer (i.e., positively charged amine functional group in EDA) adsorbed on the negatively charged abrasive-surface slightly increased with the EDA concentration. However, the secondary abrasive size increase by adding EDA would not influence greatly the PI-film polishing rate, since the variation of the secondary size was ~ 50 nm. Thus, the drastic dependency of the PI-film polishing rate of the EDA concentration was mainly related to the chemical effect of EDA, showing that the PI-film polishing rate increased linearly with the EDA concentration. In addition, the zeta potential of the colloidal silica abrasives was negatively enhanced from − 50 to − 72 mV as soon as the EDA of 7.5 wt% was added in a PI-film CMP slurry and then was enhanced slightly and negatively with further EDA concentration increase. This result indicates that the zeta potential of the colloidal silica abrasives does not influence the PI-film polishing-rate during CMP. This is because a higher negatively charged zeta potential leads to a higher repulsive force between the negatively charged PI-film-surface and the negatively charged colloidal silica abrasive surface. In general, a higher repulsive force results in a lower PI-film polishing-rate due to a lower rubbing force between the PI-film and the colloidal silica abrasive surface^31,32^. The slurry pH enhanced significantly from 9.2 to 11.7 upon the addition of 7.5 wt% EDA to a PI-film CMP slurry. Furthermore, the pH slightly increased from 11.7 to 12.24 when the EDA concentration increased from 7.5 to 17.5 wt%. Thus, the OH^−^ mole concentration in the PI-film CMP slurry rapidly increased from 0.00002 to 0.005 upon the addition of 7.5 wt% EDA to the PI-film CMP slurry, and further increase in the EDA concentration increased the OH^−^ mole concentration in a log-scale manner. This result indicates that a shift toward a higher pH by increasing the EDA concentration enhances the PI-film polishing-rate, since a higher OH^−^ concentration in a PI-film slurry can accelerate the hydrolysis reaction on the film surface^33,34^. It is noteworthy that a hydrolysis reaction between a PI-film-surface and OH^−^ ions in the PI-film CMP slurry can transform the PI-film-surface into a film surface containing pyromellitic dianhydride (PMDA) and 4,4′-oxydianiline (ODA); thus, hydrolysis reaction of a PI-film-surface can create a soft PI-film-surface. As discussed above, an increase in the OH^−^ concentration in a PI-film CMP slurry by adding EDA dominantly increases the PI-film polishing-rate by enhancing the hydrolysis reaction. To further confirm this, the dependency of the PI-film polishing-rate on the pH of the CMP slurry without adding EDA was tested, where the pH of the CMP slurry was enhanced by adding the titrant KOH, as shown in Fig. 2b. When the pH of the CMP slurry increased from 11.7 to 12.24, the PI-film polishing-rate without adding EDA slightly increased from 545 to 689 nm/min. However, the polishing-rate upon adding EDA significantly increased from 1314 to 1975 nm/min. This result shows that the hydrolysis reaction enhanced through OH^−^-ion concentration enhancement upon the addition of EDA to a PI-film CMP slurry slightly increased the PI-film polishing-rate. In contrast, the enhancement of another hydrolysis reaction by adding EDA in the slurry remarkably enhances the PI-film-surface. Thus, the linear increase in the PI-film polishing-rate with respect to the EDA concentration in the slurry is associated with the hydrolysis reaction between the PI-film-surface and OH^−^ ions, as well as another hydrolysis reaction on the PI-film-surface.

### Dependency of the decomposition degree via hydrolysis reactions on the concentration of the hydrolysis reaction accelerator EDA in the PI-film CMP slurry

**Figure 3:**
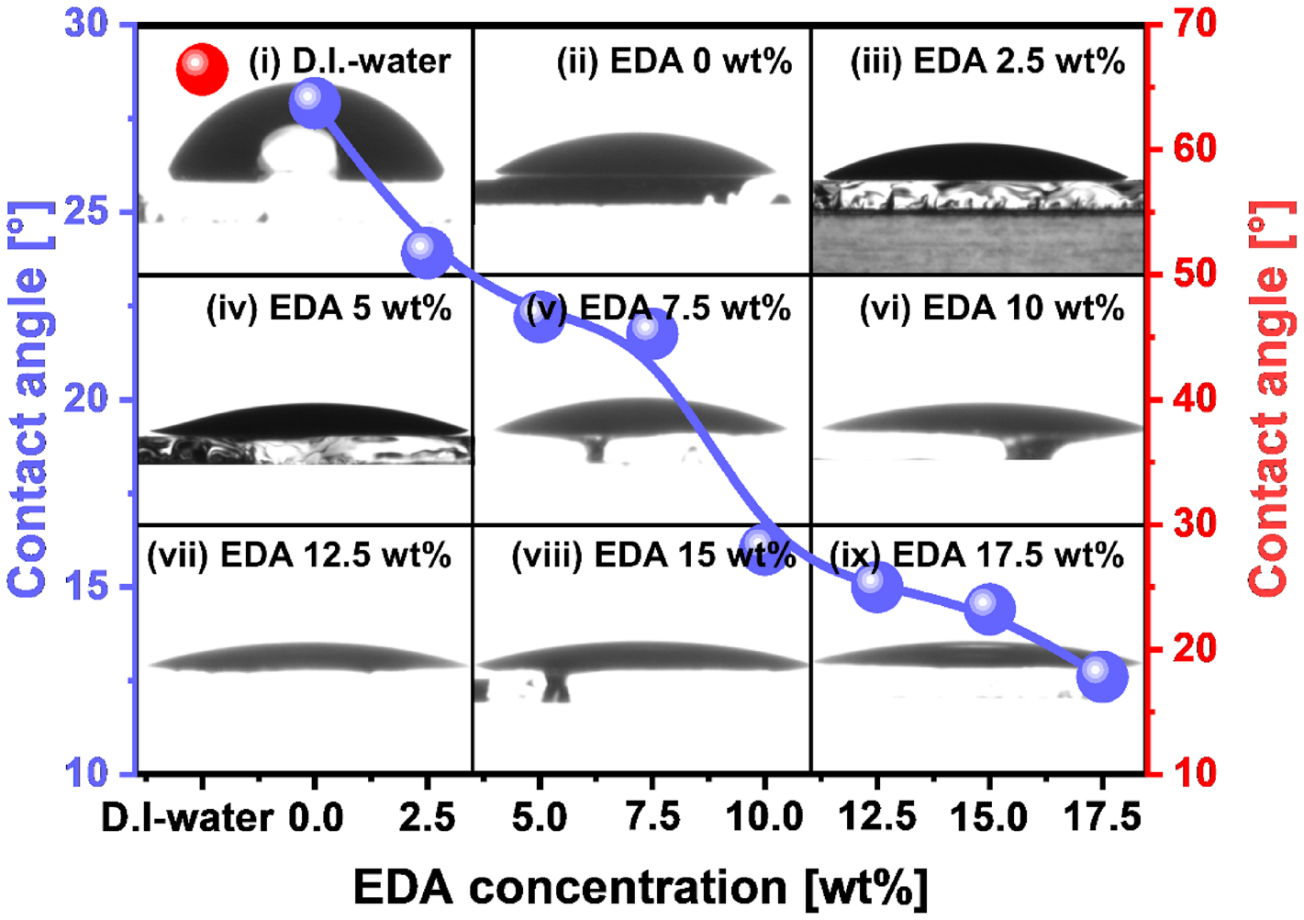
Slurry adsorption degree (i.e., contact angle) on the PI-film-surface depending on the EDA concentration in the PI-film CMP slurry. Contact angles after dropping (i) DI water, (ii) slurry without EDA, and slurries with (iii) 7.5 wt%, (iv) 10 wt%, (v) 12.5 wt%, (vi) 15.0 wt%, and (vii) 17.5 wt% EDA.

The dependence of the adsorption degree on the EDA concentration was analyzed by measuring the slurry contact angle on the PI-film-surface. This measurement was performed to find another hydrolysis reaction for the enhancement of the PI-film polishing-rate during the CMP in the presence of a hydrolysis reaction accelerator containing amine functional group (EDA). It is noteworthy that a higher degree of slurry adsorption on the PI-film-surface could result in an enhanced hydrolysis reaction between hydrolysis reaction accelerator and the PI-film-surface. The DI water contact angle on the PI-film-surface was 66.4°, where the contact angle was measured by dropping 0.01 mL of DI water on the PI-film-surface, as shown by the red circle in Fig. 3(i). This result indicates that the PI-film-surface was remarkably hydrophobic, and the zeta potential of the PI-film-surface was highly negatively charged (− 63 mV), as shown in Fig. S3. In addition, the slurry contact angle without EDA was 27.9°, where the contact angle on the PI-film-surface was measured by dropping a 0.01 mL of slurry without EDA, as shown in Fig. 3(ii). Note that the dropping of a CMP slurry rather than DI-water would not disrupt contact angle because of a repulsive force between negatively charged colloidal silica abrasives in the CMP slurry and the negative charge PI-film surface. The contact angle of the slurry without EDA (27.9°) was significantly lower than that of DI water (66.4°), which is associated with the slurry pH (~ 9.2) and the presence of 3 wt% negatively charged colloidal silica abrasives (particle size = 350 nm) in the slurry. On the other hand, for the slurries with EDA, the slurry contact angle on the PI-film-surface notably and gradually decreased from 27.9° to 12.6° when the increase in the EDA concentration from 0 to 17.5 wt%, as shown in Fig. 3(ii)–(ix). This result shows that the PI-film-surface becomes significantly more hydrophilic with the increase in the EDA concentration in the slurry, that is, a higher EDA concentration in the slurry leads to a lower slurry contact angle (i.e., higher hydrophilicity). Thus, the addition of EDA to the slurry enhanced the degree of adsorption of the slurry on the PI-film-surface during CMP. Because EDA is composed of two amine functional groups covalently bonded with ethane-1,2-diamine, the functional groups could be highly positively charged in the slurry at a strong alkaline pH (R-NH_3_^+^). The highly positively charged R-NH_3_^+^ could be adsorbed on the highly negatively charged PI-film-surface, thereby increasing the adsorption degree of the slurry on the PI-film-surface with respect to the EDA concentration.

**Figure 4:**
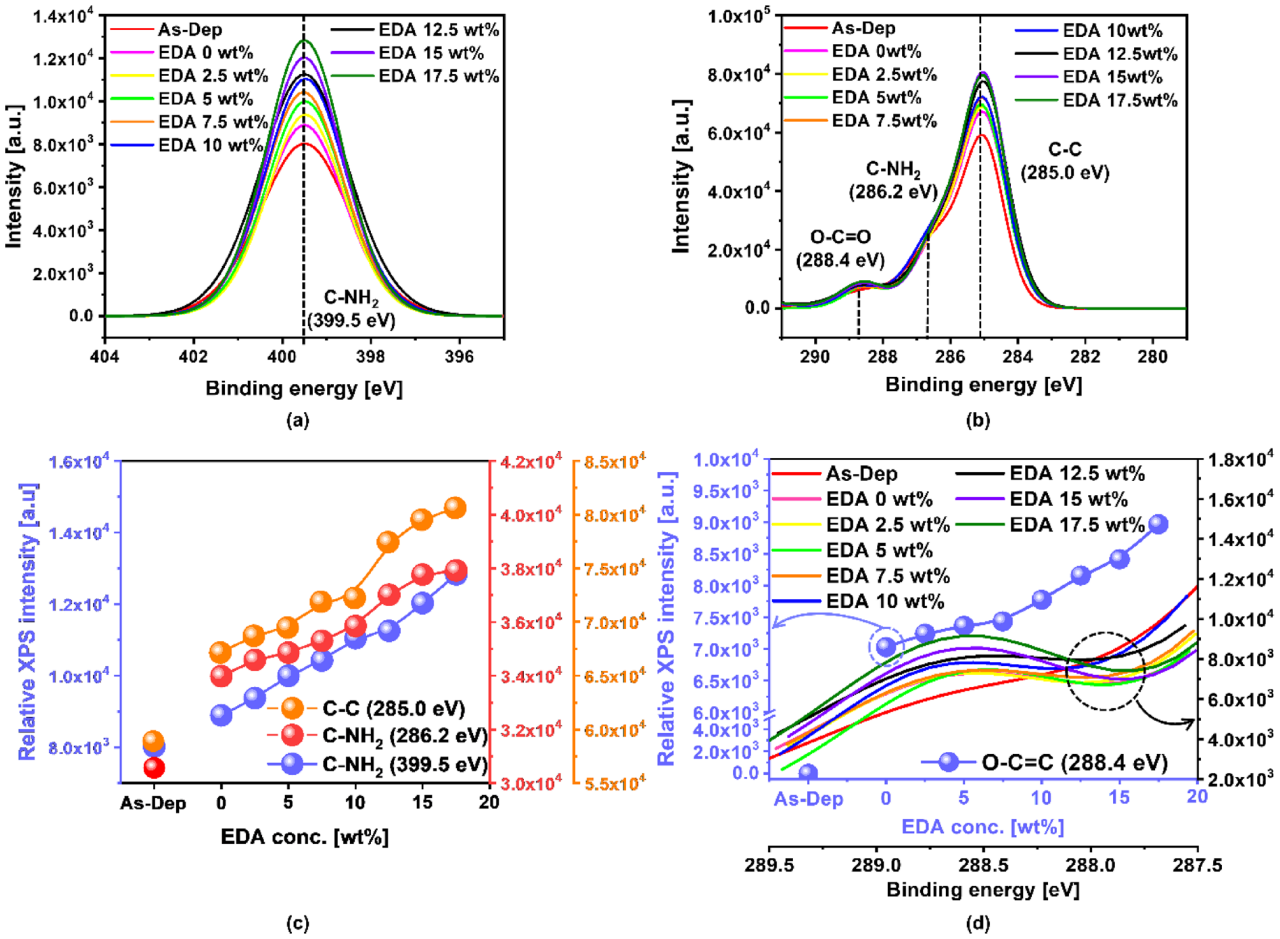
Changes in the chemical composition of the PI-film-surface as a function of the EDA concentration in the CMP slurry after CMP. XPS peaks of (**a**) C–NH_2_ at 399.5 eV, (**b**) O–C=O at 288.4 eV, C–NH_2_ at 286.2 eV, and C–C at 285.0 eV. (**c**) Dependencies of relative XPS peak intensity on EDA concentration for C–C at 285.0 eV, C–NH_2_ at 286.2 eV, and C–NH_2_ at 399.5 eV. (**d**) Dependencies of the relative XPS intensity on binding energy and the EDA concentration for the O–C=O peak at 288.4 eV.

The chemical compositions of the PI-film-surface immediately after CMP using the slurries with EDA were analyzed by XPS as a function of the EDA concentration in the slurry. This analysis was performed to further confirm the dependency of the slurry adsorption degree on the EDA concentration and to understand how the slurry adsorption on the PI-film-surface accelerates the hydrolysis reaction on the PI-film-surface. The XPS peaks of C–C, C–NH_2_ (carbon-bonding-based), and C–NH_2_ (nitrogen bonding) bonds were observed at 285.0, 286.2, and 399.5 eV, as shown in Fig. 4a,b. All chemical bonds on the PI-film-surface almost linearly increased with the EDA concentration in the slurry; however, the higher sequence of relative XPS peak intensity was followed by C–C, C–NH_2_ (carbon-bonding-based), and C–NH_2_ (nitrogen bonding), as shown in Fig. 4c. This result evidently proves that positively charged amine functional groups of EDA in the slurry are well adsorbed on the PI-film-surface during CMP. In addition, the adsorption degree of R-NH_2_ on the PI-film-surface increases linearly with the EDA concentration in the slurry during CMP, which is well correlated with the dependency of the slurry contact angle on the EDA concentration in the slurry (Fig. 3). The XPS peak of the carboxyl functional groups (i.e., O–C=O) of the PI-film-surface after CMP was located at 288.4 eV, as shown in Fig. 4d. As a reference, for the PI-film-surface without CMP, the XPS peak of O–C=O was not found at 288.4 eV, as shown by the **red** line in Fig. 4d, indicating that the thermally cured PI-film-surface was not chemically decomposed at room temperature. On the other hand, for the PI-film-surface after CMP using the slurry without EDA at pH 9.2, the relative XPS peak intensity of O–C=O (i.e., 7228 a.u.) was evidently found, as shown by the **pink** line in Fig. 4d. This implies that the OH^−^ ions in a highly alkaline slurry can result in a hydrolysis reaction to chemically decompose the thermally cured PI-film-surface into the film surface containing PMDA and ODA. It should be noted that a higher degree of hydrolysis reaction on the PI-film-surface during CMP can lead to a higher PI-film polishing-rate. Moreover, for the PI-film-surface after CMP using the slurry with EDA, the relative XPS intensity of O–C=O almost linearly and significantly increased from 7433.9 to 8965.7 a.u. with the increase in the EDA concentration up to 7.5 wt%, as shown in **other** color (i.e., orange, blue, black, purple, and green) lines of Fig. 4d. This observation proves that the presence of EDA in the CMP slurry during CMP can remarkably accelerate the hydrolysis reaction to chemically decompose the thermally cured PI-film-surface into PMDA and ODA. In particular, the degree of the hydrolysis reaction increased almost linearly with the EDA concentration in the CMP slurry during CMP. As a result, the PI-film polishing-rate increased evidently, through the hydrolysis reaction induced by additional EDA, with respect to the EDA concentration in the slurry, as shown in Figs. 1b, 2b. The relationship between the relative XPS peak intensity of the PI-film-surface with carboxyl functional groups and the EDA concentration exhibited the following: the increase in the PI-film polishing-rate via the hydrolysis reaction induced by adding EDA in the slurry was noticeably higher than that via the hydrolysis reaction induced by using only OH^−^ ions, as shown in Figs. 2b, 4d.

**Figure 5:**
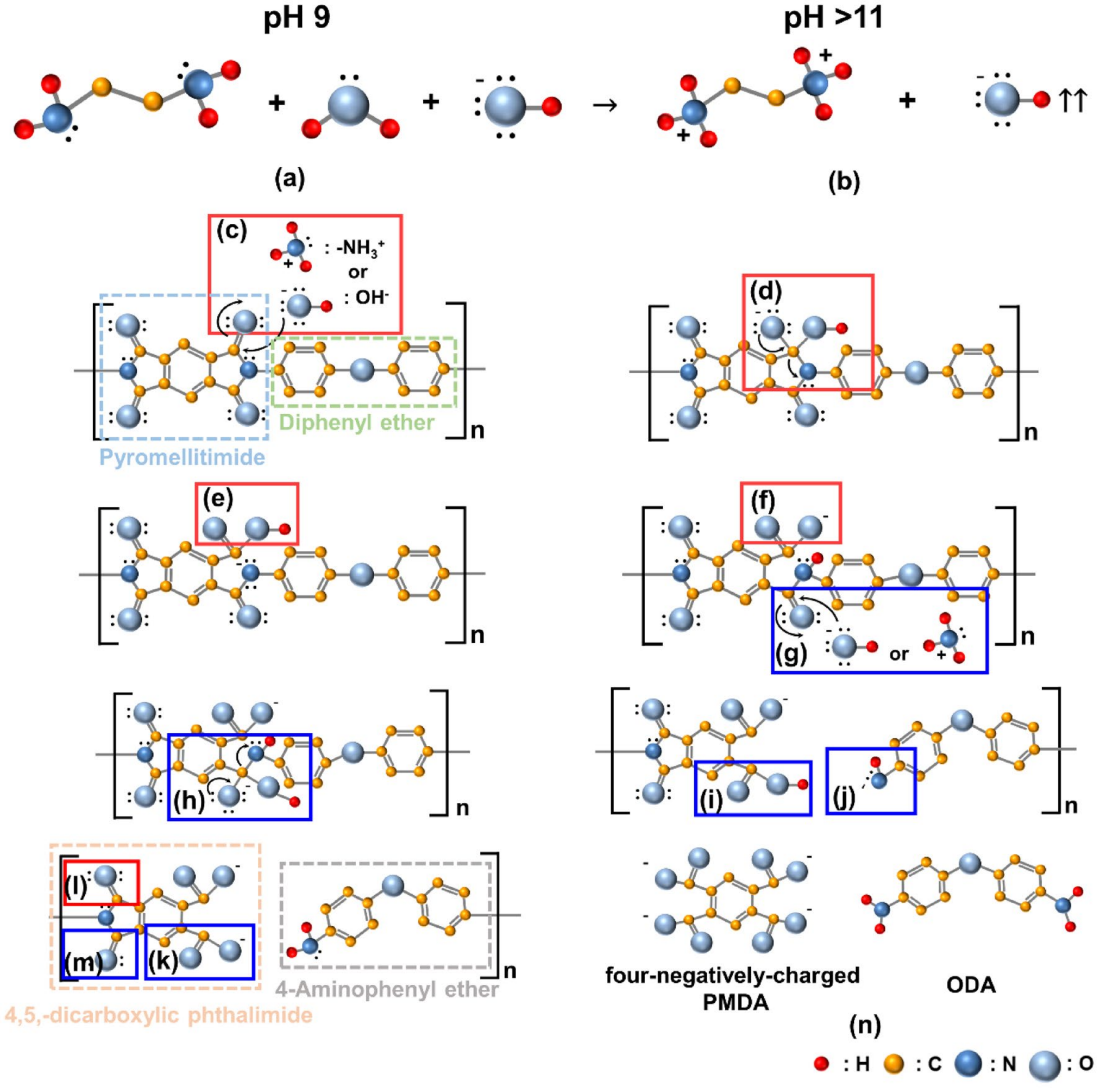
Chemical decomposition mechanism of a hard PI-film-surface into a soft PI-film-surface (i.e., four-negatively charged PMDA, and ODA) during a CMP using the slurry including a hydrolysis reaction accelerator.

The adsorption degree of the positively charged amine functional groups of EDA on the PI-film-surface and the degree of chemical decomposition from PI to PMDA and ODA via a hydrolysis reaction increased significantly with the EDA concentration in the slurry. Therefore, the mechanism by which the addition of EDA in the slurry enhanced the PI-film polishing-rate could be understood by considering the following processes: (1) adsorption of EDA in the slurry on the PI-film-surface, (2) chemical decomposition of the PI-film-surface via hydrolysis reactions between OH^−^ ions or positive-charged amine functional groups of EDA and the PI-film-surface, and (3) mechanical rubbing between colloidal silica abrasives and the chemically decomposed PI-film-surface during CMP. Polyimide is a polymer composed of pyromellitimide covalently bonded with diphenyl ether, as shown in Fig. 5c. Moreover, polyimide is a strongly covalent-bonded polymer after thermal curing (i.e., hardness of 0.33 GPa) and its surface is extremely difficult to be polished and etched, resulting in a notably low polishing-rate of ~ 500 nm/min, as shown in Fig. 1b. It should be noted that the PI-film corresponds to polyimide cured by thermal treatment. Thus, the chemical decomposition of the PI-film-surface into PMDA and ODA should be accompanied to enhance the PI-film polishing-rate during CMP^33–35^. In our experimental results, it was found that the presence of OH^−^ ions or the positively charged amine functional groups of EDA in the slurry enhanced the polishing-rate of the PI-film-surface during CMP. In addition, the presence of OH^−^ ions or the positively charged amine-functional groups of EDA in the slurry generated carboxyl functional groups on the PI-film-surface after CMP. This indicates that the presence of both OH^−^ ions and positively charged amine functional groups would induce a chemical decomposition of the PI-film-surface into PMDA and ODA, consequently enhancing the PI-film polishing-rate. This influence of OH^−^ ions and positively charged amine functional groups was further reviewed in detail.

### Mechanism of PI-film polishing-rate enhancement by adding EDA

The addition of EDA in the slurry being chemically-composed of H_2_O, OH^-^ ions, and the negatively charged (i.e., ~ -50 mV) colloidal-silica-abrasives (i.e., pH 9.2) produced noticeably the positive charged amine functional groups of EDA via a hydrolysis reaction generating a high concentration of OH^-^ ions (i.e., > pH 11.0), as shown in Fig. 5a, b. In particular, a higher concentration of EDA led to a higher concentration of OH^−^ ion and a more negative zeta potential of the colloidal silica abrasives in the slurry, as shown in Fig. 2a. Thus, the slurry included the positively charged amine functional groups of EDA, OH^−^ ions, and negatively charged colloidal silica abrasives. During CMP, as soon as the slurry without EDA (as a reference) was dropped and adsorbed on the initially neutral PI-film-surface, the zeta potential of the PI-film-surface became significantly negative (> − 60 mV), resulting in a pH of 9.2, as shown in Fig. S3. In addition, when the slurry with EDA was dropped on the highly negatively charged PI-film-surface during CMP, the adsorption degree of the slurry on the film surface was significantly enhanced. This is because the positively charged amine functional groups of EDA in the slurry were diffused and adsorbed on the film surface via an attractive Columbic reaction between amine groups and the negatively charged PI-film-surface, presenting a hydrophilic slurry contact angle of < 21.8°, as shown in Fig. 3. Thereafter, two types of hydrolysis reactions occurred that chemically decomposed the PI-film-surface into the film surface containing PMDA and ODA. These hydrolysis reactions are: (1) between OH^−^ ions and the oxygen atoms covalently bonded to the pyromellitimide of polyimide and (2) between the positively charged amine functional groups of EDA and the oxygen atoms covalently bonded to the pyromellitimide of polyimide. The hydrolysis reactions followed seven sequential chemical reaction steps. A hydrolysis reaction is a complicated substitution-nucleophilic-bimolecular reaction (i.e., the SN2 reaction). First, the OH^−^ ion or the positively charged amine functional groups of EDA reacted with the oxygen atoms covalently bonded to pyromellitimide, providing two unshared electrons to the oxygen atoms and transforming them into negatively charged oxygen atoms. These oxygen atoms were covalently bonded to pyromellitimide and OH, as shown in Fig. 5c,d. Second, two unshared electrons with the single-covalent-bonded oxygen atoms transferred into nitrogen atoms such that the a singly-negatively charged nitrogen atoms were de-bonded and the single-covalent-bonded oxygen atoms with two unshared electrons and OH were transformed into neutral carboxyl functional groups, which is called a ring-opening reaction, as shown in Fig. 5d,e. Third, the hydrogen atoms in the neutral carboxyl functional groups were transferred into the negatively nitrogen atoms so that the neutral carboxyl functional groups were transformed into negatively charged carboxyl functional groups and the nitrogen atoms were changed to neutral nitrogen atoms, which is called proton transfer, as shown in Fig. 5e,f. Fourth, the chemical reaction shown in Fig. 5g–h followed the chemical reaction shown in Fig. 5c,d. Fifth, the chemical reaction in Fig. 5h, i is similar to that in Fig. 5d,e, which shows that diphenyl ether with negatively charged nitrogen atoms was de-bonded from 4,5,-dicarboxylic phthalimide. Finally, the chemical reaction in Fig. 5j,k was performed according to the chemical reaction shown in Fig. 5e,f, producing 4,5,-dicarboxylic phthalimide and 4-Aminophenyl ether. When these seven sequential chemical reaction steps were repeated with two double-covalent-bonded oxygen atoms in Fig. 5l,m, negatively charged (i.e., − 4) PMDA and neutral ODA were produced, as shown in Fig. 5. Therefore, the hydrolysis reaction between OH^−^ ions or positively charged amine functional groups of EDA and the PI-film-surface chemically decomposed the PI-film-surface into a soft film surface containing negatively charged PMDA and neutral ODA, which was confirmed by the presence of carboxyl functional groups on the PI-film-surface after CMP (Fig. 4d). Thus, the addition of EDA in the slurry could enhance the PI-film polishing-rate via mechanical rubbing between the negatively charged colloidal silica abrasives and the chemically decomposed PI-film-surface (i.e., a film surface containing negatively charged PMDA and neutral ODA). Moreover, a higher EDA concentration in the slurry presented a higher XPS peak intensity of the carboxyl functional groups on the PI-film-surface after CMP, as shown in Fig. 4b. Therefore, a higher EDA concentration in the slurry would result in a higher degree of chemical decomposition of the PI-film-surface into PMDA and ODA. As a result, a higher EDA concentration in the slurry resulted in a higher PI-film polishing-rate.

## Discussion

To achieve a high polishing-rate of a thermally cured PI-film-surface, the surface chemical-structure deformation via hydrolysis should occur during CMP, which can produce a soft PI-film-surface because the thermally cured PI-film has a hard PI-film-surface. The PI-film CMP slurry, including a strong hydrolysis reaction accelerator, was designed by adding EDA to the CMP slurry with colloidal silica abrasives. The addition of neutral EDA to alkaline DI water readily dissociated DI water into OH^−^ ions and R-NH_3_^+^ (i.e., a positively charged EDA); thus, a higher EDA concentration in the slurry led to both OH^−^ ions and R-NH_3_^+^ concentration increased. During the CMP of the PI-film-surface, two types of hydrolysis reactions were conducted to chemically decompose a harder PI-film-surface to a soft PI-film-surface. These reactions include a hydrolysis reaction between the OH^−^ or R-NH_3_^+^ ions and oxygen atoms in the PI-film-surface, and the reactions were performed using the CMP slurry with EDA. In particular, the degree of slurry adsorption on the PI-film-surface was significantly enhanced with the increase in the EDA concentration in the CMP slurry, because the positively charged amine functional groups of EDA in the CMP slurry reacted attractively with the PI-film-surface. Thus, the hydrolysis reaction probability between the CMP slurry and PI-film-surface increased noticeably and linearly with respect to the EDA concentration in the slurry. In addition, the presence of two types of hydrolysis reactions using the slurry was confirmed by the presence of carboxyl functional groups on the PI-film-surface after CMP. This is a clear evidence of the chemical decomposition of the PI-film-surface into negatively charged PMDA and neutral ODA via hydrolysis reactions during CMP. The presence of OH^−^ ions and R-NH_3_^+^ in the slurry significantly enhanced the relative XPS peak intensity of the carboxyl functional groups. Hence, the relative XPS peak intensity of the carboxyl functional group concentration increased linearly and significantly with increasing EDA concentration. By chemically decomposing the PI-film-surface into PMDA and ODA, the PI-film polishing-rate could be increased surprisingly by enhancing a chemically dominant CMP rather than a mechanical-dominant CMP of the PI-film-surface. Thus, the PI-film polishing-rate increased significantly with increasing EDA concentration in the slurry. The addition of a hydrolysis reaction accelerator (i.e., small molecules with amine functional groups) in the CMP slurry can noticeably enhance the chemically dominant CMP ability. Therefore, the design of abrasives in the CMP slurry, such as abrasive materials (i.e., colloidal silica, ceria, and zirconia abrasives), and the abrasive shape and size would be necessary to further enhance the PI-film polishing-rate by multiplying the mechanically dominant CMP ability. In addition, after CMP, the design of the abrasive zeta potential in the slurry would essentially reduce the adsorption of the remaining abrasives and polishing-induced residues to minimize the polishing induced scratches.

## Methods

### Materials

A 3000-nm-thick polyimide (PI) film was coated on a glass substrate and thermally cured. The hardness of the PI-film was 0.33 GPa, as shown in Fig. S1. 350-nm colloidal silica abrasives were synthesized at 25 °C for 4 h using ethanol (C_2_H_5_OH, DEAJUNG), ammonia solution (NH_4_OH, Junsei Chemical), tetraethyl orthosilicate (SiC_8_H_2_OO_4_, RAM tech), and deionized (DI) water. Ethanol of 608.12 g, ammonia solution of 252.29 g, and DI of 642.52 g were first mixed into the reactor, and then stirring was performed for 1 min. After that, tetraethyl orthosilicate of 150 g was dropped at 10 g/min, and synthesis was performed for 4 h. at 25 °C. Thereafter, the colloidal silica abrasives were centrifuged twice at 8000 rpm for 10 min. Then, slurries were produced using 3 wt% of colloidal silica abrasives, 0–17.5 wt% of a hydrolysis reaction accelerator (EDA; C_2_H_8_N_2_; Sigma Aldrich), and DI water. Notably, the pH of the slurry containing only colloidal silica abrasives was 9.2, and that of the slurry containing both the colloidal silica abrasives and EDA increased from 11.7 to 12.2 with the increase in the EDA concentration from 7.5 to 17.5 wt%.

### Chemical–mechanical planarization

The 3000-nm-thick PI-film coated on glass was cut into a 4 cm × 4 cm square. The CMP of the PI-film-surface was conducted using a CMP polisher (POLI-300, G&P Tech. Inc., Korea) implanted with a rectangular-grooved CMP pad (SUBA 400, Nitta Haas Inc., Japan). A pad break-in for warming the polishing was performed with a brush conditioner for 10 min, and then two dummy wafers were polished prior to the main polishing of the PI-film-surface. Ex situ pad conditioning was conducted after each polishing for the various slurries. The applied head pressure was 4 psi, the rotation speed of the carrier holding the PI-film samples was 80 rpm/min, and the rotation speed of the table attached to the CMP pad was 80 rpm/min. The flow rate of the CMP slurry was fixed at 100 mL/min, and the polishing time was set to 60 s. After 1 min of CMP, all the PI-film samples were buffed with DI water to eliminate the remaining abrasives on the PI-film-surface. The presence of the remaining abrasives on the PI-film-surface was investigated using an optical microscope, as shown in Fig. S2.

### Characterization

The polishing-rate of the PI-film was estimated by measuring the film thickness before and after the CMP using an ellipsometry (V-VASE, J.A. Woollam Co., Inc.). The secondary size and zeta potential of the colloidal silica abrasives in the CMP slurry were analyzed using a particle analyzer (ELSZ2 + , Otsuka Electronics). The polishing-rate of the Ti films was calculated by measuring the sheet resistance of the Ti film before and after CMP using a four-point probe (CMTSR5000, AIT). Scanning electron microscopy (SEM, S-4800, Hitachi) was performed on the synthesized colloidal silica abrasives at an acceleration voltage of 15 kV. The pH and conductivity of the PI-film CMP slurries were measured using a pH meter (Thermo Scientific, ORIONSTAR A211). The contact angle was measured using a contact angle meter (GBX Instrument, DIGIDROP) by dropping 0.01 mL of DI water or slurries on the film surface. The chemical composition of the PI-film-surface after CMP was characterized using XPS (K-Alpha + , Thermo Fisher Scientific) at 12 keV and 6 mA with an Al Kα (1486.6 eV).

## Supplementary Information

Supplementary Information.

## Data Availability

The datasets generated during and/or analyzed during the current study are available from the corresponding author on reasonable request.
