# Supplementary material for: Polymer link breakage of polyimide-film-surface using hydrolysis reaction accelerator for enhancing chemical–mechanical-planarization polishing-rate
Source: Sci Rep. 2022 Mar 1;12:3366. doi: 10.1038/s41598-022-07340-y (PMC8888717; doi:10.1038/s41598-022-07340-y)
Supplement: Supplementary file 1 — Supplementary Information. [file 41598_2022_7340_MOESM1_ESM.docx]

**Supplementary Figures**

**Chemical Decomposition of Thermally Cured Polyimide-Film-Surface into Charged Pyromellitic dianhydride and Neutral 4,4’-oxydianiline to Remarkably Enhance the Chemical–Mechanical-Planarization Polishing-Rate**

*Gi-Ppeum Jeong^1^, Jun-Seong Park^1^, Seung-Jae Lee^1^, Pil-su Kim^2^, Man-Hyup Han^2^, Seong-Wan Hong^2^, Eun-Seong Kim^2^, Jin-Hyung Park^3^, Byoung-Kwon Choo^4^, Seung-Bae Kang^4^ and Jea-Gun Park^1, 2^**

**Figure S1**


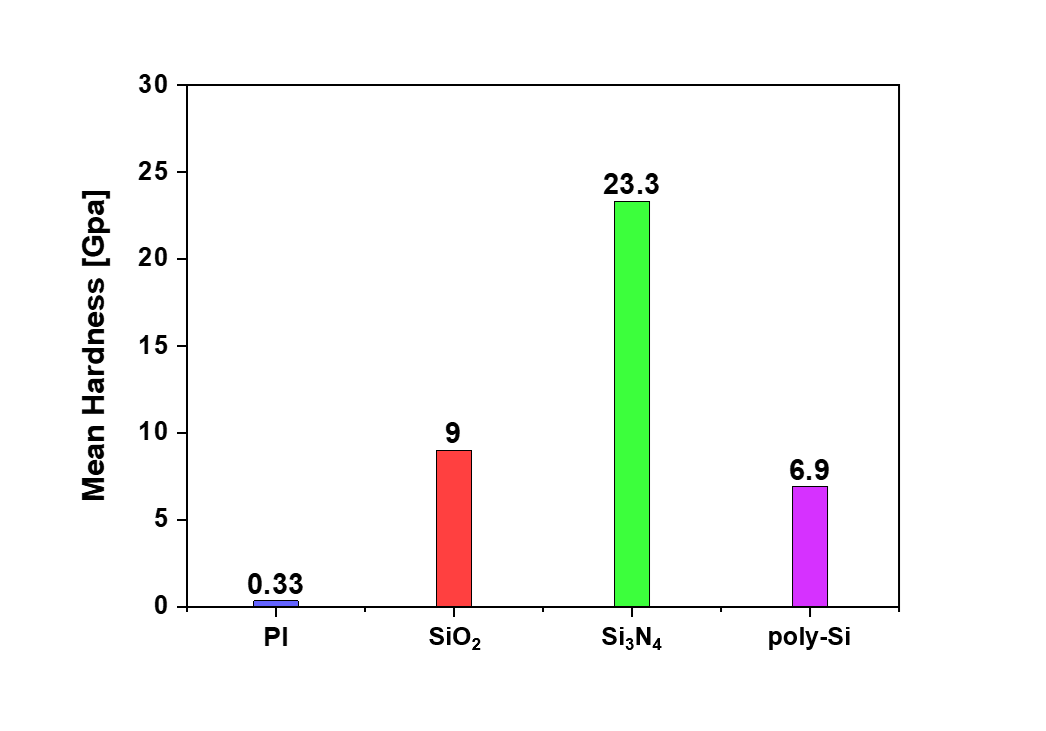


**Figure. S1**. Hardness of PI-film-surface for PI-, SiO­_2_-, Si_3_N_4_-, poly-Si-film surface. The higher sequence of hardness was followed by Si_3_N_4_-, SiO_2_­-, poly-Si-, and PI-film-surface.

**Figure S2**

**
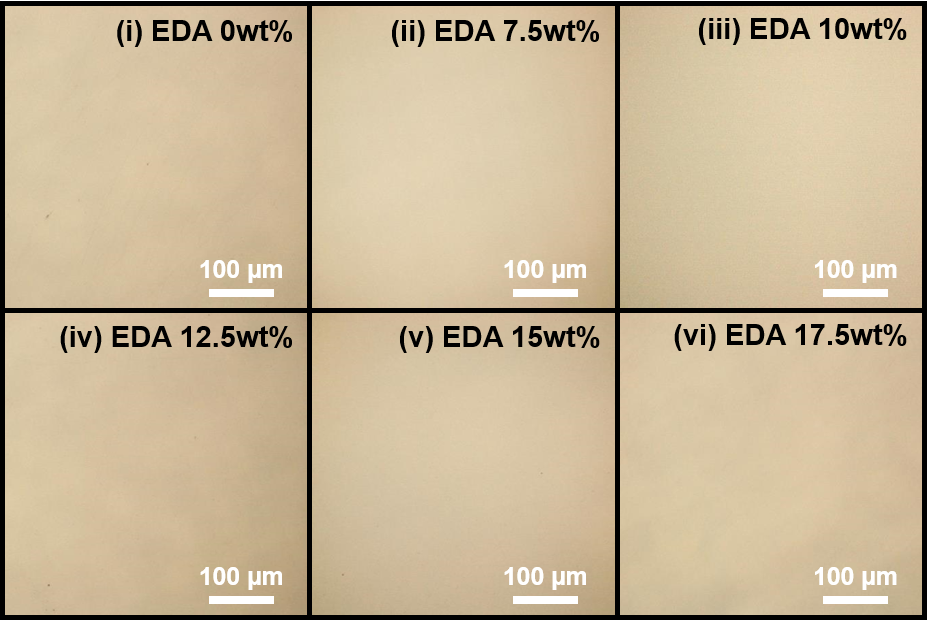
**

**Figure. S2**. Optical microscope images of PI-film-surface after a CMP using the slurry including a hydrolysis reaction accelerator (i.e., EDA) of (i) 0-, (ii) 7.5-, (iii) 10-, (iv) 12.5-, (v) 15-, and (vi) 17.5-wt%. All PI-film-surface after a CMP showed none of remaining colloidal-silica-abrasives due to a strong repulsive force between highly negative charged colloidal-silica-abrasives and a highly negative charged PI-film-surface, where a CMP process was conducted by a CMP for 60 s and D.I buffing for 60 s.

**Figure S3**


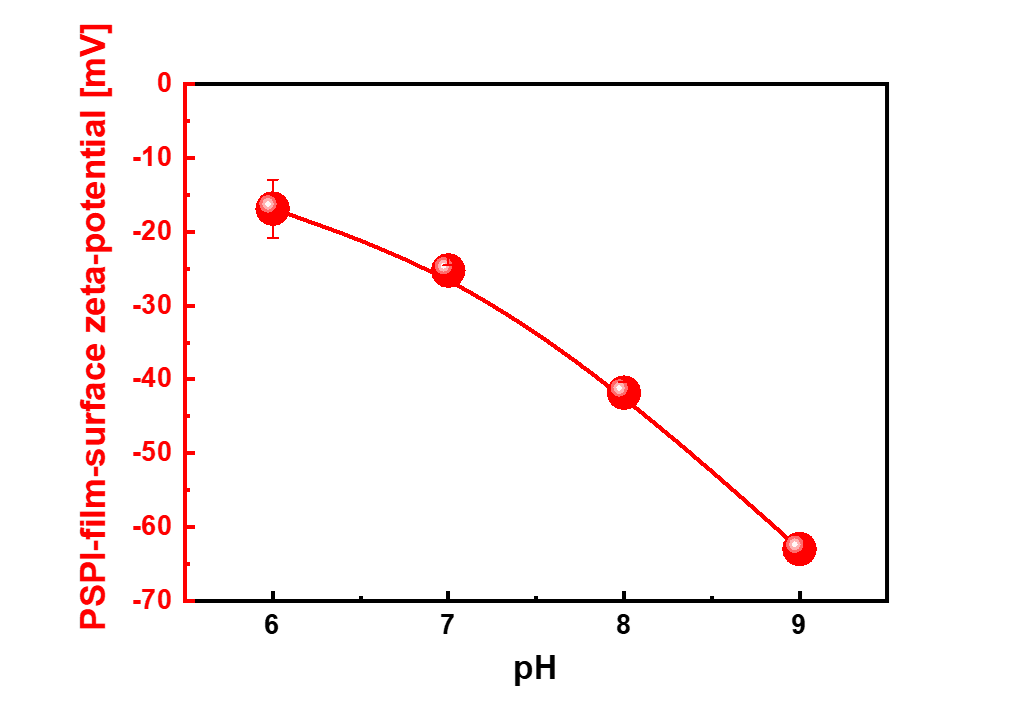


**Figure. S3**. Dependency of PI-film-surface zeta-potential on the slurry pH. The PI-film-surface zeta-potential decreased remarkably with increasing pH. The PI-film-surface presented a high negative zeta-potential at pH 9 (i.e., **-**63 mV). Thus, the PI-film-surface zeta-potential would be much higher negative zeta-potential above pH 9 (i.e., < **-**63 mV).
